# 1-Ethyl-4-{2-[1-(4-methyl­phen­yl)ethyl­idene]hydrazinyl­idene}-3,4-dihydro-1*H*-2λ^6^,1-benzothia­zine-2,2-dione

**DOI:** 10.1107/S1600536813000202

**Published:** 2013-01-09

**Authors:** Muhammad Shafiq, M. Nawaz Tahir, William T. A. Harrison, Tanveer Hussain Bokhari, Muhammad Safder

**Affiliations:** a Department of Chemistry, Government College University, Faisalabad 38000, Pakistan; b Department of Physics, University of Sargodha, Sargodha, Pakistan; c Department of Chemistry, University of Aberdeen, Mston Walk, Aberdeen AB24 3UE, Scotland

## Abstract

In the title compound, C_19_H_21_N_3_O_2_S, the dihedral angle between the aromatic rings is 6.7 (2)° and the C=N—N=C torsion angle is 178.0 (2)°. The conformation of the thia­zine ring is an envelope, with the S atom displaced by 0.802 (2) Å from the mean plane of the other five atoms (r.m.s. deviation = 0.022 Å). In the crystal, mol­ecules are linked by C—H⋯O inter­actions, generating *C*(5) chains propagating in [010]. A weak C—H⋯π inter­action is also observed.

## Related literature

For the synthesis and biological activity of the title compound and related materials, see: Shafiq *et al.* (2011*a* ▶). For further synthetic details, see: Shafiq *et al.* (2011*b* ▶). For a related structure, see: Shafiq *et al.* (2013 ▶).
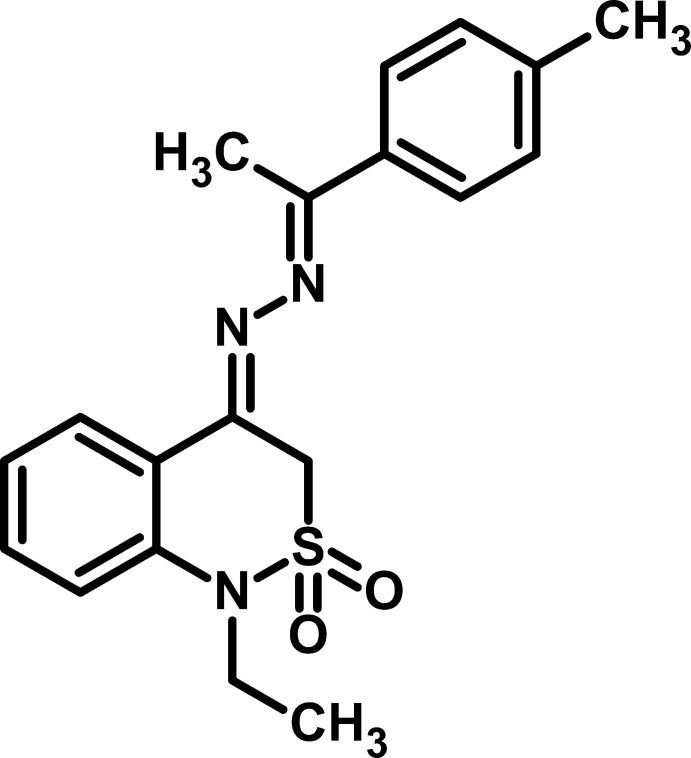


## Experimental

### 

#### Crystal data

C_19_H_21_N_3_O_2_S
*M* *_r_* = 355.45
Monoclinic, 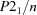

*a* = 15.9018 (10) Å
*b* = 7.3716 (4) Å
*c* = 16.8376 (10) Å
β = 111.644 (3)°
*V* = 1834.57 (19) Å^3^
*Z* = 4
Mo *K*α radiation
μ = 0.19 mm^−1^
*T* = 296 K
0.34 × 0.26 × 0.24 mm

#### Data collection

Bruker APEXII CCD diffractometer
Absorption correction: multi-scan (*SADABS*; Bruker, 2007 ▶) *T*_min_ = 0.937, *T*_max_ = 0.955
14168 measured reflections
3597 independent reflections
2598 reflections with *I* > 2σ(*I*)
*R*_int_ = 0.025

#### Refinement

*R*[*F* ^2^ > 2σ(*F* ^2^)] = 0.048
*wR*(*F* ^2^) = 0.139
*S* = 1.02
3597 reflections
229 parameters
H-atom parameters constrained
Δρ_max_ = 0.45 e Å^−3^
Δρ_min_ = −0.40 e Å^−3^

### 

Data collection: *APEX2* (Bruker, 2007 ▶); cell refinement: *SAINT* (Bruker, 2007 ▶); data reduction: *SAINT*; program(s) used to solve structure: *SHELXS97* (Sheldrick, 2008 ▶); program(s) used to refine structure: *SHELXL97* (Sheldrick, 2008 ▶); molecular graphics: *ORTEP-3* (Farrugia, 2012 ▶); software used to prepare material for publication: *SHELXL97*.

## Supplementary Material

Crystal structure: contains datablock(s) global, I. DOI: 10.1107/S1600536813000202/ld2091sup1.cif

Structure factors: contains datablock(s) I. DOI: 10.1107/S1600536813000202/ld2091Isup2.hkl

Supplementary material file. DOI: 10.1107/S1600536813000202/ld2091Isup3.cml

Additional supplementary materials:  crystallographic information; 3D view; checkCIF report

## Figures and Tables

**Table 1:** Hydrogen-bond geometry (Å, °) *Cg*3 is the centroid of the C13–C18 ring.

*D*—H⋯*A*	*D*—H	H⋯*A*	*D*⋯*A*	*D*—H⋯*A*
C7—H7*A*⋯O2^i^	0.97	2.50	3.402 (4)	155
C9—H9*A*⋯*Cg*3^ii^	0.97	2.67	3.613 (2)	165

Symmetry codes: (i) 
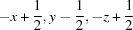
; (ii) 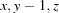.

**Fig. 1.**
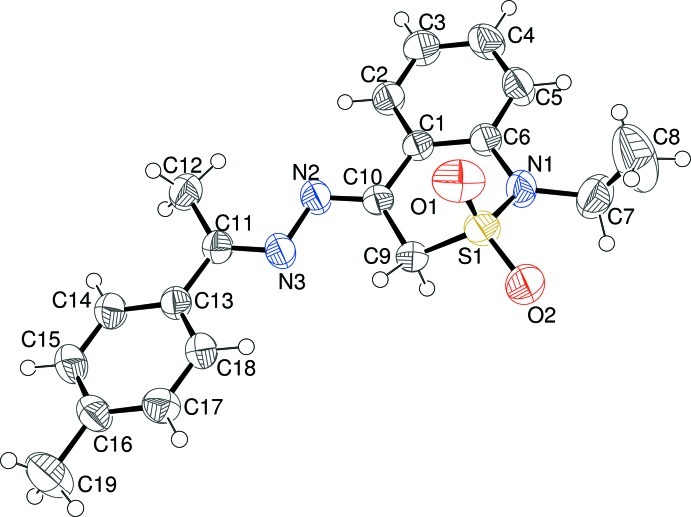
The molecular structure of (I), showing displacement ellipsoids at the 50% probability level.

## supplementary crystallographic information

### Comment

As a part of our ongoing studies of benzothiazine derivatives with potential biactivity (Shafiq *et al.*, 2011*a*,*b*), we now describe the synthesis and structure of the title compound, (I).

The dihedral angle between the C1–C6 and C13–C18 aromatic rings is 6.68 (15)° and the C10=N2—N3=C11 torsion angle is 177.98 (19)°. The conformation of the C1/C6/C9/C10/N1/S1 thiazine ring is an envelope, with the S atom displaced by 0.802 (2) Å from the mean plane of the other five atoms (r.m.s. deviation = 0.022 Å). A very similar conformation was observed in a related structure (Shafiq *et al.*, 2013). Atoms C7 and C8 in (I) are displaced from the mean plane of the thiazine ring by -0.416 (5) and 0.704 (6) Å, respectively.

In the crystal, the moelcules are linked by C—H···O interactions (Table 1) to generate C(5) chains propagating in the *b* axis direction. A weak C—H···π interaction to the C13—C18 ring also occurs (Table 1).

### Experimental

4-Hydrazinylidene-1-ethyl-3*H*-2?6,1-benzothiazine-2,2-dione (Shafiq *et al.*, 2011*b*) was reacted with *para*-methyl acetophenone according to literature procedure (Shafiq, *et al.*, 2011*a*). The product obtained was re-crystallized from ethyl acetate solution to yield yellow prisms.

### Refinement

The H atoms were placed in calculated positions (C—H = 0.93–0.97 Å) and refined as riding. The methyl group was allowed to rotate, but not to tip, to best fit the electron density. The constraint *U*_iso_(H) = 1.2*U*_eq_(C) or 1.5*U*_eq_(methyl C) was applied.

### Crystal data

**Table:**

C_19_H_21_N_3_O_2_S	*F*(000) = 752
*M**_r_* = 355.45	*D*_x_ = 1.287 Mg m^−^^3^
Monoclinic, *P*2_1_/*n*	Mo *K*α radiation, λ = 0.71073 Å
Hall symbol: -P 2yn	Cell parameters from 335 reflections
*a* = 15.9018 (10) Å	θ = 3.1–23.5°
*b* = 7.3716 (4) Å	µ = 0.19 mm^−^^1^
*c* = 16.8376 (10) Å	*T* = 296 K
β = 111.644 (3)°	Prism, yellow
*V* = 1834.57 (19) Å^3^	0.34 × 0.26 × 0.24 mm
*Z* = 4	

### Data collection

**Table:**

Bruker APEXII CCD diffractometer	3597 independent reflections
Radiation source: fine-focus sealed tube	2598 reflections with *I* > 2σ(*I*)
Graphite monochromator	*R*_int_ = 0.025
ω scans	θ_max_ = 26.0°, θ_min_ = 1.5°
Absorption correction: multi-scan (*SADABS*; Bruker, 2007)	*h* = −19→18
*T*_min_ = 0.937, *T*_max_ = 0.955	*k* = −9→8
14168 measured reflections	*l* = −18→20

### Refinement

**Table:**

Refinement on *F*^2^	Primary atom site location: structure-invariant direct methods
Least-squares matrix: full	Secondary atom site location: difference Fourier map
*R*[*F*^2^ > 2σ(*F*^2^)] = 0.048	Hydrogen site location: inferred from neighbouring sites
*wR*(*F*^2^) = 0.139	H-atom parameters constrained
*S* = 1.02	*w* = 1/[σ^2^(*F*_o_^2^) + (0.0649*P*)^2^ + 0.6783*P*] where *P* = (*F*_o_^2^ + 2*F*_c_^2^)/3
3597 reflections	(Δ/σ)_max_ = 0.001
229 parameters	Δρ_max_ = 0.45 e Å^−^^3^
0 restraints	Δρ_min_ = −0.40 e Å^−^^3^

### Special details

**Table:**

Geometry. All e.s.d.'s (except the e.s.d. in the dihedral angle between two l.s. planes) are estimated using the full covariance matrix. The cell e.s.d.'s are taken into account individually in the estimation of e.s.d.'s in distances, angles and torsion angles; correlations between e.s.d.'s in cell parameters are only used when they are defined by crystal symmetry. An approximate (isotropic) treatment of cell e.s.d.'s is used for estimating e.s.d.'s involving l.s. planes.
Refinement. Refinement of *F*^2^ against ALL reflections. The weighted *R*-factor *wR* and goodness of fit *S* are based on *F*^2^, conventional *R*-factors *R* are based on *F*, with *F* set to zero for negative *F*^2^. The threshold expression of *F*^2^ > σ(*F*^2^) is used only for calculating *R*-factors(gt) *etc*. and is not relevant to the choice of reflections for refinement. *R*-factors based on *F*^2^ are statistically about twice as large as those based on *F*, and *R*- factors based on ALL data will be even larger.

### Fractional atomic coordinates and isotropic or equivalent isotropic displacement parameters (Å^2^)

**Table:**

	*x*	*y*	*z*	*U*_iso_*/*U*_eq_	
C1	0.53653 (13)	0.1072 (3)	0.23121 (12)	0.0401 (5)	
C2	0.62165 (14)	0.1299 (3)	0.22572 (14)	0.0504 (6)	
H2	0.6306	0.2260	0.1940	0.060*	
C3	0.69200 (16)	0.0146 (4)	0.26570 (16)	0.0610 (7)	
H3	0.7478	0.0324	0.2609	0.073*	
C4	0.67962 (16)	−0.1279 (4)	0.31306 (17)	0.0629 (7)	
H4	0.7274	−0.2057	0.3411	0.076*	
C5	0.59694 (17)	−0.1552 (3)	0.31882 (16)	0.0609 (7)	
H5	0.5891	−0.2528	0.3504	0.073*	
C6	0.52430 (14)	−0.0399 (3)	0.27836 (14)	0.0460 (5)	
C7	0.4152 (2)	−0.2572 (4)	0.3072 (2)	0.0795 (9)	
H7A	0.3511	−0.2787	0.2768	0.095*	
H7B	0.4483	−0.3488	0.2894	0.095*	
C8	0.4360 (3)	−0.2746 (6)	0.3986 (3)	0.1315 (16)	
H8A	0.4972	−0.2366	0.4293	0.197*	
H8B	0.4290	−0.3989	0.4121	0.197*	
H8C	0.3954	−0.1999	0.4147	0.197*	
C9	0.37033 (14)	0.2050 (3)	0.18892 (14)	0.0478 (5)	
H9A	0.3372	0.1275	0.1410	0.057*	
H9B	0.3380	0.3191	0.1820	0.057*	
C10	0.46398 (13)	0.2394 (3)	0.18915 (12)	0.0411 (5)	
C11	0.43384 (14)	0.6397 (3)	0.08403 (13)	0.0451 (5)	
C12	0.52607 (16)	0.6781 (3)	0.08357 (17)	0.0603 (6)	
H12A	0.5486	0.7891	0.1138	0.090*	
H12B	0.5226	0.6896	0.0257	0.090*	
H12C	0.5661	0.5804	0.1110	0.090*	
C13	0.36016 (14)	0.7729 (3)	0.04561 (13)	0.0443 (5)	
C14	0.36872 (17)	0.9137 (3)	−0.00555 (15)	0.0558 (6)	
H14	0.4221	0.9255	−0.0157	0.067*	
C15	0.30017 (17)	1.0357 (4)	−0.04138 (15)	0.0620 (7)	
H15	0.3081	1.1287	−0.0753	0.074*	
C16	0.21992 (17)	1.0238 (3)	−0.02836 (14)	0.0553 (6)	
C17	0.21100 (16)	0.8847 (3)	0.02377 (14)	0.0532 (6)	
H17	0.1578	0.8748	0.0344	0.064*	
C18	0.27955 (15)	0.7613 (3)	0.05990 (13)	0.0497 (5)	
H18	0.2719	0.6691	0.0943	0.060*	
C19	0.1445 (2)	1.1567 (5)	−0.0695 (2)	0.0903 (10)	
H19A	0.1632	1.2757	−0.0467	0.135*	
H19B	0.0920	1.1212	−0.0580	0.135*	
H19C	0.1301	1.1580	−0.1302	0.135*	
S1	0.37561 (4)	0.10183 (9)	0.28376 (4)	0.0544 (2)	
O1	0.42132 (12)	0.2209 (3)	0.35183 (11)	0.0773 (6)	
O2	0.28939 (11)	0.0333 (3)	0.27645 (13)	0.0771 (6)	
N1	0.43901 (13)	−0.0742 (3)	0.28385 (14)	0.0600 (6)	
N2	0.48467 (12)	0.3786 (3)	0.15443 (12)	0.0536 (5)	
N3	0.41244 (13)	0.4972 (3)	0.11660 (12)	0.0546 (5)	

### Atomic displacement parameters (Å^2^)

**Table:**

	*U*^11^	*U*^22^	*U*^33^	*U*^12^	*U*^13^	*U*^23^
C1	0.0382 (11)	0.0417 (12)	0.0417 (10)	0.0006 (9)	0.0160 (8)	−0.0016 (9)
C2	0.0434 (12)	0.0530 (14)	0.0597 (13)	0.0020 (10)	0.0248 (10)	0.0066 (11)
C3	0.0432 (13)	0.0687 (17)	0.0777 (16)	0.0086 (11)	0.0300 (12)	0.0084 (14)
C4	0.0482 (14)	0.0678 (17)	0.0753 (16)	0.0203 (12)	0.0257 (12)	0.0186 (13)
C5	0.0576 (15)	0.0597 (16)	0.0716 (15)	0.0152 (12)	0.0311 (12)	0.0236 (13)
C6	0.0406 (12)	0.0484 (13)	0.0526 (12)	0.0046 (9)	0.0214 (9)	0.0069 (10)
C7	0.0672 (18)	0.071 (2)	0.109 (2)	−0.0089 (14)	0.0431 (17)	0.0118 (17)
C8	0.167 (4)	0.132 (4)	0.138 (3)	0.049 (3)	0.105 (3)	0.063 (3)
C9	0.0376 (11)	0.0468 (13)	0.0555 (12)	0.0009 (9)	0.0132 (9)	0.0071 (10)
C10	0.0383 (11)	0.0425 (12)	0.0425 (10)	0.0002 (9)	0.0146 (9)	0.0005 (9)
C11	0.0472 (12)	0.0440 (13)	0.0459 (11)	−0.0017 (9)	0.0194 (9)	0.0027 (10)
C12	0.0517 (14)	0.0525 (15)	0.0792 (16)	−0.0029 (11)	0.0272 (12)	0.0070 (13)
C13	0.0477 (12)	0.0415 (12)	0.0425 (10)	−0.0024 (9)	0.0150 (9)	0.0020 (9)
C14	0.0538 (14)	0.0563 (15)	0.0589 (13)	−0.0025 (11)	0.0227 (11)	0.0149 (11)
C15	0.0665 (16)	0.0588 (16)	0.0601 (14)	0.0018 (12)	0.0226 (12)	0.0227 (12)
C16	0.0605 (15)	0.0522 (14)	0.0467 (12)	0.0083 (11)	0.0122 (11)	0.0093 (11)
C17	0.0489 (13)	0.0597 (15)	0.0511 (12)	0.0041 (11)	0.0186 (10)	0.0026 (11)
C18	0.0528 (13)	0.0472 (13)	0.0492 (12)	−0.0005 (10)	0.0191 (10)	0.0074 (10)
C19	0.083 (2)	0.099 (2)	0.089 (2)	0.0348 (18)	0.0322 (17)	0.0373 (19)
S1	0.0401 (3)	0.0653 (4)	0.0638 (4)	0.0066 (3)	0.0262 (3)	0.0137 (3)
O1	0.0671 (12)	0.1064 (16)	0.0636 (10)	0.0069 (10)	0.0302 (9)	−0.0119 (10)
O2	0.0449 (10)	0.0878 (13)	0.1101 (15)	0.0094 (9)	0.0422 (10)	0.0334 (11)
N1	0.0461 (11)	0.0570 (13)	0.0836 (14)	0.0062 (9)	0.0316 (10)	0.0266 (11)
N2	0.0456 (11)	0.0509 (12)	0.0679 (12)	0.0070 (8)	0.0251 (9)	0.0179 (10)
N3	0.0481 (11)	0.0499 (12)	0.0687 (12)	0.0076 (9)	0.0249 (9)	0.0193 (10)

### Geometric parameters (Å, º)

**Table:**

C1—C6	1.399 (3)	C11—N3	1.287 (3)
C1—C2	1.400 (3)	C11—C13	1.482 (3)
C1—C10	1.477 (3)	C11—C12	1.497 (3)
C2—C3	1.368 (3)	C12—H12A	0.9600
C2—H2	0.9300	C12—H12B	0.9600
C3—C4	1.376 (3)	C12—H12C	0.9600
C3—H3	0.9300	C13—C14	1.387 (3)
C4—C5	1.368 (3)	C13—C18	1.391 (3)
C4—H4	0.9300	C14—C15	1.370 (3)
C5—C6	1.393 (3)	C14—H14	0.9300
C5—H5	0.9300	C15—C16	1.375 (3)
C6—N1	1.416 (3)	C15—H15	0.9300
C7—C8	1.456 (5)	C16—C17	1.391 (3)
C7—N1	1.493 (3)	C16—C19	1.505 (3)
C7—H7A	0.9700	C17—C18	1.377 (3)
C7—H7B	0.9700	C17—H17	0.9300
C8—H8A	0.9600	C18—H18	0.9300
C8—H8B	0.9600	C19—H19A	0.9600
C8—H8C	0.9600	C19—H19B	0.9600
C9—C10	1.509 (3)	C19—H19C	0.9600
C9—S1	1.742 (2)	S1—O1	1.4135 (19)
C9—H9A	0.9700	S1—O2	1.4233 (17)
C9—H9B	0.9700	S1—N1	1.643 (2)
C10—N2	1.282 (3)	N2—N3	1.397 (2)
			
C6—C1—C2	118.04 (19)	C11—C12—H12A	109.5
C6—C1—C10	122.43 (18)	C11—C12—H12B	109.5
C2—C1—C10	119.52 (19)	H12A—C12—H12B	109.5
C3—C2—C1	121.9 (2)	C11—C12—H12C	109.5
C3—C2—H2	119.1	H12A—C12—H12C	109.5
C1—C2—H2	119.1	H12B—C12—H12C	109.5
C2—C3—C4	119.6 (2)	C14—C13—C18	117.4 (2)
C2—C3—H3	120.2	C14—C13—C11	121.6 (2)
C4—C3—H3	120.2	C18—C13—C11	120.97 (19)
C5—C4—C3	120.0 (2)	C15—C14—C13	121.4 (2)
C5—C4—H4	120.0	C15—C14—H14	119.3
C3—C4—H4	120.0	C13—C14—H14	119.3
C4—C5—C6	121.4 (2)	C14—C15—C16	121.5 (2)
C4—C5—H5	119.3	C14—C15—H15	119.3
C6—C5—H5	119.3	C16—C15—H15	119.3
C5—C6—C1	119.1 (2)	C15—C16—C17	117.7 (2)
C5—C6—N1	120.1 (2)	C15—C16—C19	120.9 (2)
C1—C6—N1	120.82 (18)	C17—C16—C19	121.4 (2)
C8—C7—N1	112.2 (3)	C18—C17—C16	121.2 (2)
C8—C7—H7A	109.2	C18—C17—H17	119.4
N1—C7—H7A	109.2	C16—C17—H17	119.4
C8—C7—H7B	109.2	C17—C18—C13	120.9 (2)
N1—C7—H7B	109.2	C17—C18—H18	119.6
H7A—C7—H7B	107.9	C13—C18—H18	119.6
C7—C8—H8A	109.5	C16—C19—H19A	109.5
C7—C8—H8B	109.5	C16—C19—H19B	109.5
H8A—C8—H8B	109.5	H19A—C19—H19B	109.5
C7—C8—H8C	109.5	C16—C19—H19C	109.5
H8A—C8—H8C	109.5	H19A—C19—H19C	109.5
H8B—C8—H8C	109.5	H19B—C19—H19C	109.5
C10—C9—S1	110.92 (14)	O1—S1—O2	118.78 (12)
C10—C9—H9A	109.5	O1—S1—N1	110.96 (11)
S1—C9—H9A	109.5	O2—S1—N1	106.91 (11)
C10—C9—H9B	109.5	O1—S1—C9	107.98 (12)
S1—C9—H9B	109.5	O2—S1—C9	110.88 (10)
H9A—C9—H9B	108.0	N1—S1—C9	99.65 (11)
N2—C10—C1	117.42 (19)	C6—N1—C7	121.4 (2)
N2—C10—C9	123.51 (19)	C6—N1—S1	117.47 (16)
C1—C10—C9	119.07 (18)	C7—N1—S1	119.90 (17)
N3—C11—C13	115.83 (19)	C10—N2—N3	113.76 (18)
N3—C11—C12	124.8 (2)	C11—N3—N2	113.78 (19)
C13—C11—C12	119.40 (19)		
			
C6—C1—C2—C3	−0.8 (3)	C15—C16—C17—C18	−1.1 (3)
C10—C1—C2—C3	177.9 (2)	C19—C16—C17—C18	178.7 (2)
C1—C2—C3—C4	−0.2 (4)	C16—C17—C18—C13	0.3 (3)
C2—C3—C4—C5	1.0 (4)	C14—C13—C18—C17	0.5 (3)
C3—C4—C5—C6	−0.8 (4)	C11—C13—C18—C17	−179.7 (2)
C4—C5—C6—C1	−0.3 (4)	C10—C9—S1—O1	61.17 (18)
C4—C5—C6—N1	178.5 (2)	C10—C9—S1—O2	−167.11 (16)
C2—C1—C6—C5	1.1 (3)	C10—C9—S1—N1	−54.73 (18)
C10—C1—C6—C5	−177.7 (2)	C5—C6—N1—C7	−19.6 (4)
C2—C1—C6—N1	−177.7 (2)	C1—C6—N1—C7	159.1 (2)
C10—C1—C6—N1	3.6 (3)	C5—C6—N1—S1	147.7 (2)
C6—C1—C10—N2	173.5 (2)	C1—C6—N1—S1	−33.5 (3)
C2—C1—C10—N2	−5.2 (3)	C8—C7—N1—C6	92.8 (3)
C6—C1—C10—C9	−6.5 (3)	C8—C7—N1—S1	−74.3 (3)
C2—C1—C10—C9	174.75 (19)	O1—S1—N1—C6	−57.8 (2)
S1—C9—C10—N2	−144.76 (19)	O2—S1—N1—C6	171.25 (17)
S1—C9—C10—C1	35.3 (2)	C9—S1—N1—C6	55.80 (19)
N3—C11—C13—C14	−167.5 (2)	O1—S1—N1—C7	109.8 (2)
C12—C11—C13—C14	13.1 (3)	O2—S1—N1—C7	−21.2 (2)
N3—C11—C13—C18	12.7 (3)	C9—S1—N1—C7	−136.6 (2)
C12—C11—C13—C18	−166.7 (2)	C1—C10—N2—N3	−179.62 (17)
C18—C13—C14—C15	−0.7 (3)	C9—C10—N2—N3	0.4 (3)
C11—C13—C14—C15	179.6 (2)	C13—C11—N3—N2	−178.06 (18)
C13—C14—C15—C16	−0.1 (4)	C12—C11—N3—N2	1.3 (3)
C14—C15—C16—C17	1.0 (4)	C10—N2—N3—C11	177.98 (19)
C14—C15—C16—C19	−178.8 (3)		

### Hydrogen-bond geometry (Å, º)

Cg3 is the centroid of the C13–C18 ring.

**Table:**

*D*—H···*A*	*D*—H	H···*A*	*D*···*A*	*D*—H···*A*
C7—H7*A*···O2^i^	0.97	2.50	3.402 (4)	155
C9—H9*A*···*Cg*3^ii^	0.97	2.67	3.613 (2)	165

Symmetry codes: (i) −*x*+1/2, *y*−1/2, −*z*+1/2; (ii) *x*, *y*−1, *z*.
